# Chronic rhinosinusitis with nasal polyps in the elderly

**DOI:** 10.1186/2045-7022-3-S2-P35

**Published:** 2013-07-16

**Authors:** Marjolein Cornet, Wytske Fokkens

**Affiliations:** 1 AMC, Amsterdam, the Netherlands; 2 AMC, Otorhinolaryngology, Amsterdam, the Netherlands

## Background

To determine the influence of age on the long term results of functional endoscopic sinus surgery (FESS) in patients with chronic rhinosinusitis with nasal polyps (CRSwNP).

## Methods

In this study we sent all adult patients who received FESS because of CRSwNP between the year 2000 and 2005 in the AMC in Amsterdam a quality of life questionnaire in the year 2012. The Lund-Mackay score was calculated for each patient. We compared postoperative subjective improvement using the SNOT questionnaire. Two different age groups were analysed separately, group 1 (age 18-65) and group 2 (over 65 years).

## Results

Response rate was 67% (151 out of 225). 104 patients were < 65 years old (group 1) and 47 patients > 65 years (group 2). The mean age at the time of questionnaire of the whole group was 56 years old (range 28-84). Mean follow-up time was 7 years (range 12 years).Of these patients 35% had a primary FESS and 65% revision surgery. There was no statistical difference in Lund-Mackay score between the two age groups. The mean postoperative total SNOT score in the whole group was 1.3. We found a tendency to significance with a lower score in the older age group compared to the younger patients (p=0.06) and in several separate SNOT domain scores we found a significant difference. The older patients scored significantly lower in the domains of ear- and head symptoms.

## Conclusion

Our subjective postoperative outcomes show a significant difference in several SNOT domains and a tendency to significance in total SNOT score between the two different age groups. These results could be influenced by a natural decrease in symptoms over time in older patients. There is still not enough evidence for this thought and further research will be needed on this subject.

